# Supplementary material for: Conditioned Media from Head and Neck Cancer Cell Lines and Serum Samples from Head and Neck Cancer Patients Drive Catabolic Pathways in Cultured Muscle Cells
Source: Cancers (Basel). 2023 Mar 19;15(6):1843. doi: 10.3390/cancers15061843 (PMC10047086; doi:10.3390/cancers15061843)
Supplement: Supplementary file 1 [file cancers-15-01843-s001.zip › cancers-2212193-supplementary.pdf]

Figure S1: Myosin blot on C2C12 cells corresponding to the results presented Figure 1E.

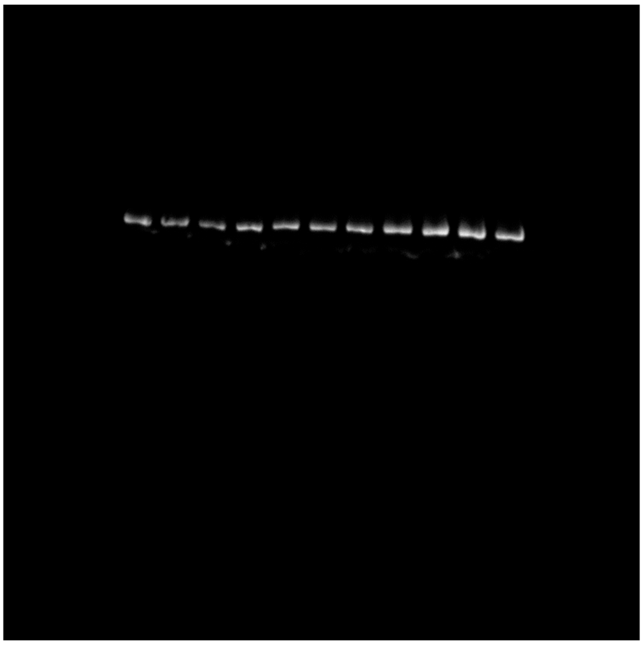

Gel9

1	2	3	4	5	6	7	8	9	10		11	12	14	15
**Echelle**	**Tp1x**	P1-	P2-	P3-	P1+	P2+	P3+	**Mix**	P1T	P2T	P3T	**Tp1x**	**Echelle**	**Tp1x**

P1-,2-, 3- are the conditions with cancer and P1T, P2T, P3T are the 3 repeats for the control condition. P1+2+3+ are conditions not presented in the paper (HPV psotive squamous cell carcinoma).

Figure S2: Blot of the fig 2 : myosin in human myotubes after incubation with different conditioned media

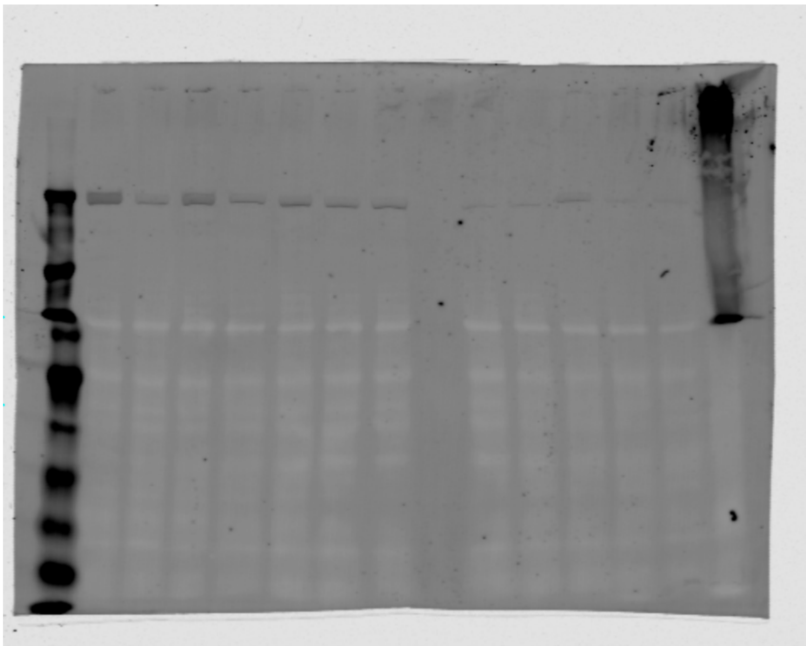

name	CMC	HPV -05	HPV -60A	Mix 1	CMC	HPV -05	HPV -60A	CMC	HPV -05	HPV -60A	CMC	HPV -05	HPV -60A
repeat	1	1	1	-	2	2	2	3	3	3	4	4	4
								no sample left					

CMC corresponds to the control.

HPV-05: to the cell line UT-SCC-5 conditioned media

HPV-60A: to the cell line UT-SCC-5 conditioned media

1-2-3-4 are the numbers of the repeat experiments.

Figure S3: Autophagy flux presented in Fig 4. In this blot we are looking at LC3B at the bottom of the blot. A cropped blot is presented in the text.

154- and + correspond to the experiments with a conditioned media made from another cell lines (UM-SCC-154) which differ by the fact that this is a human papillomavirus induced cancer cell lines. The results from the experiments with this cell line are not presented in the text.

The number 48 correspond to 48 hour of incubation with the conditioned media.

First repeat

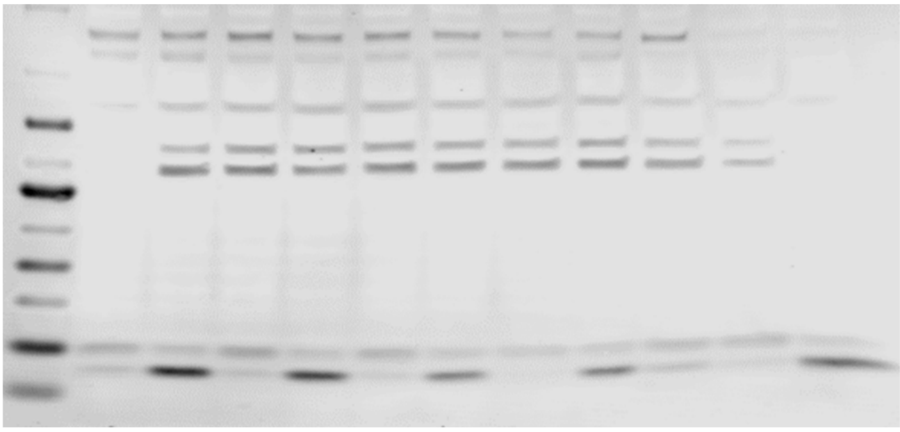

1	2	3	4	5	6	7	8	9	10	11	12
Ladder	CMC -	CMC+	5-	5+	60-	60+	154-	154+	Mix	Control Neg	Control plus

Second repeat:

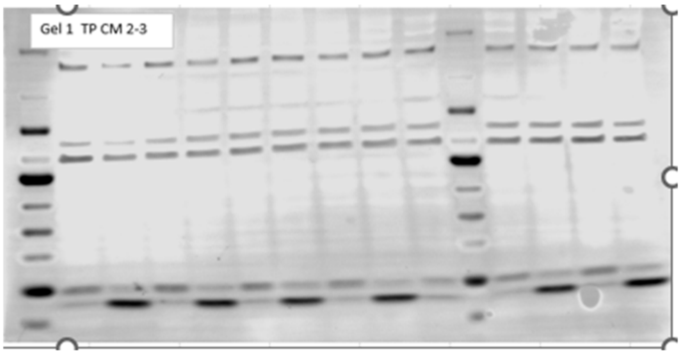

10	11	12	13	14	15
Mix	Ladder	CMC- 48	CMC+48	5 - 48	5+ 48

The results presented in the Fig 4 are from the analysis of the blot on the right of the right ladder

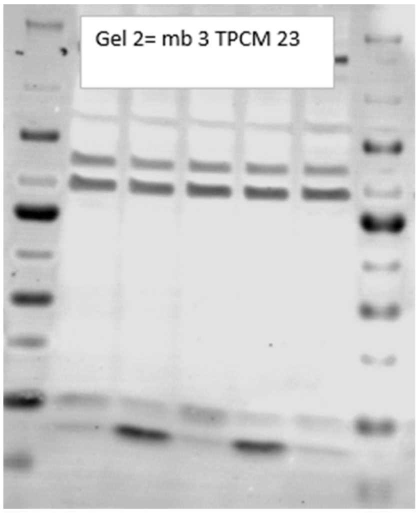

1	2	3	4	5	6	7
Ladder	60 - 48	60+ 48	154 - 48	154+ 48	Mix	Ladder

Third repeat:

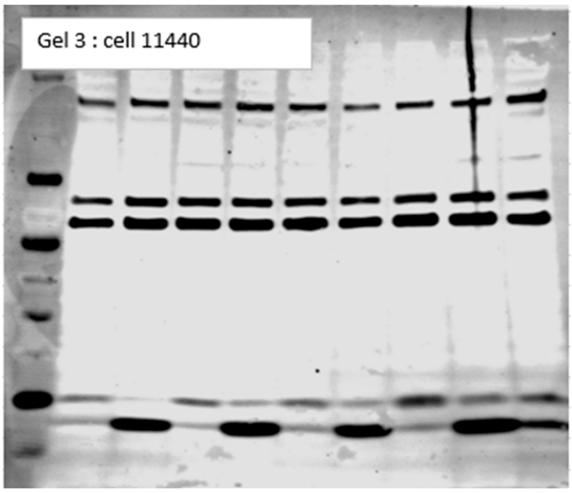

1	2	3	4	5	6	7	8	9	10
Ladder	CMC- 48	CMC+ 48	5 - 48	5+ 48	60 - 48	60+ 48	154 - 48	154+ 48	Mix

Figure S4: Myosine expression in myotubes after incubation with patient's sera conditioned media.

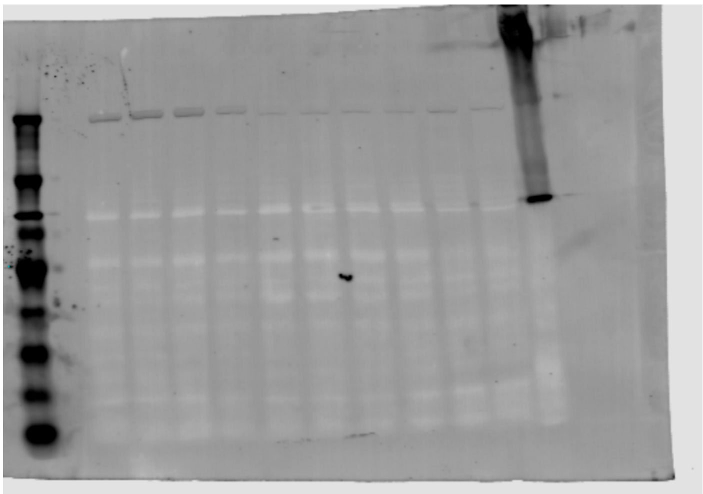

Control	Low sarco	Severe sar	intergel nc	Control	Low sarco	Severe sar	Control	Low sarco	Severe sarcopenia	
T1	B1	A1	Mix 1	T2	B2	A2	T3	B3	A3	

A: SS group mix of sera

B: MS group mix of sera

Figure S5: Autophagy flux in differentiated myotubes incubated with mix of sera from control or cancer patients (MS and SS groups) corresponding to the Fig. 7 B in the text.

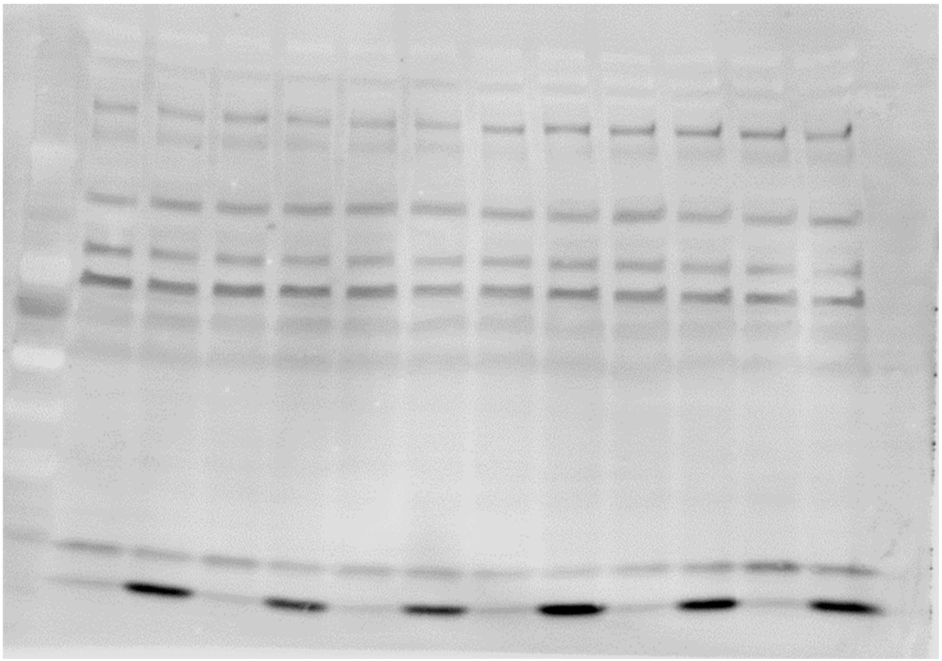

LC3 membrane

1	2	3	4	5	6	7	8	9	10	11	12	13
Ladder	A1-	A1+	B1-	B1+	T1-	T1+	A2-	A2+	B2-	B2+	T2-	T2+

T1,2: control with 2 repeats

B1,2: mild sarcopenia patient mix of sera: 2 repeats

A 1,2: severe sarcopenia patient mix of sera: 2 repeats

+ and – correspond to the condition with or without chloroquine.

Table S1: Blood parameters between patients with severe sarcopenia (SS group) or mild sarcopenia (MS group)

	SS group	MS group	p-value
C-Reactive Protein (mg/L)	39.7+/-43.7	20.6+/-29.1	0.27
Total protein(g/L)	68.2+/-6.36	70.4+/-5.2	0.41
Total cholesterolemia (g/L)	1.5+/-0.5	2.1+/-1.0	0.08
Triglyceridemia(g/L)	1.7+/-0.7	1.9+/-0.9	0.46
HDL(g/L)	0.4+/-0.1	0.6+/-0.3	0.11
TSH (mUI/L)	1.38+/-1.44	2.5+/-2.4	0.27
GDF-15 (pg/ml)	663 [457-1464]	514 [345-946]	0.47
FGF-21 (pg/ml)	208 [33-541]	177 [34-280]	0.48
Testosteronemia(nmol/L)	9.3+/-6.1	12.9+/-7.7	0.25
IL-6 (pg/ml)	37 [15-153]	42 [10-113]	0.63
IL-8 (pg/ml)	14 [6-44]	1 [0-13]	0.13

Follistatin (pg/ml)	4.7 [3.9-6.0]	4.2 [3.1-6.5]	0.61
IGF-1 (pg/ml)	1.1 [1.0-1.7]	1.3 [1.1-3.6]	0.34

Table S2: Plasma amino-acid concentration between patients with severe sarcopenia (SS group) and mild sarcopenia group (MS group). EAA: Essential amino-acids. BCAA: branch chain amino-acids and TAA: total amino-acids (TAA).

	SS group	MS group	p-value
Glutamic acid	53+/-24	75+/-23	0.06
Asparagine	40+/-10	38+/-4	0.58
Serine	91+/-31	88+/-19	0.81
Gluatamine	546+/-105	579+/-78	0.52
Histidine	67+/-18	75+/-11	0.30
Glycine	172+/-55	189+/-63	0.54
Threonine	92+/-29	85+/-18	0.59
3-methylhistidine	9+/-4	9+/-5	0.77
Citrulline	25+/-9	28+/-6	0.35
Arginine	72+/-22	62+/-15	0.29
Alanine	244+/-44	251+/-106	0.86
Taurine	43+/-21	53+/-12	0.27
Tyrosine	56+/-14	46+/-7	0.08
Valine	212+/-44	215+/-31	0.87
Methionine	20+/-5	20+/-3	0.97
Tryptophan	34+/-6	27+/-8	0.08
Phenylalanine	52+/-6	53+/-9	0.85
Isoleucine	57+/-14	60+/-7	0.53
Ornithine	51+/-10	57+/-7	0.16
Leucine	123+/-31	119+/-21	0.79
Lysine	140+/-23	143+/-18	0.82
EAA	797+/-128	798+/-82	0.99
BCAA	392+/-87	395+/-55	0.93
TAA	2200+/-351	2270+/-270	0.65
EAA/TAA ratio	0.36+/-0.03	0.35+/-0.03	0.47
